# Safety and efficacy of Hypofractionated stereotactic radiosurgery for high-grade Gliomas at first recurrence: a single-center experience

**DOI:** 10.1186/s12885-021-07856-y

**Published:** 2021-02-05

**Authors:** Yun Guan, Ji Xiong, Mingyuan Pan, Wenyin Shi, Jing Li, Huaguang Zhu, Xiu Gong, Chao Li, Guanghai Mei, Xiaoxia Liu, Li Pan, Jiazhong Dai, Yang Wang, Enmin Wang, Xin Wang

**Affiliations:** 1 CyberKnife Center, Department of Neurosurgery, Huashan Hospital, Fudan University, 12 Wulumuqi Road (M), Shanghai, 200040 China; 2 Neurosurgical Institute of Fudan University, 12 Wulumuqi Road (M), Shanghai, 200040 China; 3 Shanghai Clinical Medical Center of Neurosurgery, 12 Wulumuqi Road (M), Shanghai, 200040 China; 4 Shanghai Key Laboratory of Brain Function and Restoration and Neural Regeneration, 12 Wulumuqi Road (M), Shanghai, 200040 China; 5 Department of pathology, Huashan Hospital, Fudan University, 12 Wulumuqi Road (M), Shanghai, 200040 China; 6 Department of Radiation Oncology, Thomas Jefferson University, Philadelphia, PA USA

**Keywords:** Hypofractionated stereotactic radiosurgery, Recurrent high-grade glioma, CyberKnife, Salvage treatment

## Abstract

**Background:**

The optimal treatment for recurrent high-grade gliomas (rHGGs) remains uncertain. This study aimed to investigate the efficacy and safety of hypofractionated stereotactic radiosurgery (HSRS) as a first-line salvage treatment for in-field recurrence of high-grade gliomas.

**Methods:**

Between January 2016 and October 2019, 70 patients with rHGG who underwent HSRS were retrospectively analysed. The primary endpoint was overall survival (OS), and secondary endpoints included both progression-free survival (PFS) and adverse events, which were assessed according to Common Toxicity Criteria Adverse Events (CTCAE) version 5. The prognostic value of key clinical features (age, performance status, planning target volume, dose, use of bevacizumab) was evaluated.

**Results:**

A total of 70 patients were included in the study. Forty patients were male and 30 were female. Forty-nine had an initial diagnosis of glioblastoma (GBM), and the rest (21) were confirmed to be WHO grade 3 gliomas. The median planning target volume (PTV) was 16.68 cm^3^ (0.81–121.96 cm^3^). The median prescribed dose was 24 Gy (12–30 Gy) in 4 fractions (2–6 fractions). The median baseline of Karnofsky Performance Status (KPS) was 70 (40–90). With a median follow-up of 12.1 months, the median overall survival after salvage treatment was 17.6 months (19.5 and 14.6 months for grade 3 and 4 gliomas, respectively; *p* = .039). No grade 3 or higher toxicities was recorded. Multivariate analysis showed that concurrent bevacizumab with radiosurgery and KPS > 70 were favourable prognostic factors for grade 4 patients with HGG.

**Conclusions:**

Salvage HSRS showed a favourable outcome and acceptable toxicity for rHGG. A prospective phase II study (NCT04197492) is ongoing to further investigate the value of hypofractionated stereotactic radiosurgery (HSRS) in rHGG.

**Supplementary Information:**

The online version contains supplementary material available at 10.1186/s12885-021-07856-y.

## Introduction

The most frequently diagnosed malignant primary brain tumour in adults is high-grade glioma (HGG). In the United States [1], there are 2.96 newly diagnosed occurrences per 100,000 people per year, while the number in China is up to 5 to 8. Despite definitive primary therapy, including surgery, adjuvant chemoradiation and temozolomide-based chemotherapy [2], nearly all patients experience tumour recurrence [3], up to 90% of which is local recurrence [4].

The clinical outcome of recurrent rHGG is poor, and there is no consensus on the optimal treatments, which consist of surgery, reirradiation systemic therapy and tumour treating fields (TTFs). Surgery has been reported to have a median OS of 9.7 months, whereas less than 30% of patients could undergo the operation due to the involvement of eloquent areas and the infiltrative nature of glioma [5–7]. Systemic therapy has a median OS ranging from 6 to 9 months [8–10]. The efficacy of salvage systemic therapy is limited because of the cumulative toxicity and resistance of chemotherapy agents [11]. For TTF, the median OS reported in EF-14 was 6.6 months [12]. Radiotherapy is also known as an option for rHGG. Several small sample prospective studies reported a promising outcome and acceptable toxicity with a median OS of 12 to 12.7 months after salvage treatment [13–16].

HSRS can deliver a high radiation dose while limiting toxicity to normal tissues. CyberKnife is a radiosurgery system that allows highly conformal image-guided radiotherapy and shows a promising tumour control effect for central nervous system tumours. This study aims to determine the treatment outcome and toxicity of HSRS. To our knowledge, this is the largest cohort of rHGGs treated with HSRS as a first-line salvage treatment.

## Methods

### Eligibility criteria and endpoints

All participants provided written informed consent, and studies were approved by the local ethics committee. Between January 2016 and October 2019, patients with recurrent HGG who received salvage HSRS using CyberKnife at HuaShan Hospital were included. All patients had initially histologically confirmed World Health Organization (WHO) grade 3/4 glioma and received adjuvant external-beam radiation with concomitant temozolomide and adjuvant temozolomide. HGG recurrence was confirmed by RANO criteria and/or stereotactic brain biopsy. All patients were treated at first recurrence within the radiation field.

The primary endpoint was overall survival from the completion of salvage HSRS (OS-HSRS). The second endpoints included progression-free survival after salvage treatment and toxicity defined by CTCAE 5.0.

### Baseline evaluation and treatment delivery

HSRS was performed using the Radiosurgery System (Accuray, Sunnyvale, CA, USA). Patients were immobilized with a custom thermoplastic mask and underwent both computed tomography (CT, GE Light speed Ultra 16 Slice, USA) examinations with a slice thickness of 1.25 mm and MRI with a slice thickness of 2 mm acquired from both T1 postcontrast and T2 flair images. CT and MRI scans were then fused using the planning system for contouring.

Radiation oncologists, neurosurgeons, and radiation physicists participated in tumour delineation, planning, and dose selection. Gross tumour volume (GTV) was defined as the gadolinium-enhanced tumour on the T1-weighted series. The clinical tumour volume (CTV) was considered equal to the GTV. The planning target volume (PTV) was a uniform 2 mm expansion of the CTV, and FLAIR abnormalities were not included in the treatment volume. Multiplan (Accuray, Sunnyvale, CA, USA) software was used for inverse planning. The prescribed dose to PTV was determined according to the target volume, site, previous irradiation volume and total dose, and the interval between treatments.

The use of systemic therapy after HSRS was decided by the treating physicians. Thus, the regimens were individualized, and most commonly, bevacizumab, temozolomide or clinical trials were recommended.

### Assessment and toxicity

All patients underwent clinical and radiological follow-up every 3 m after HSRS. If any significant deterioration in the patient’s performance occurred, an MRI was ordered immediately. The radiological examination included MRI and other necessary examinations, such as MRI-based spectroscopy, perfusion MRI, and methionine positron emission tomography. KPS after treatment, adverse event occurrence, and associated clinical outcomes were recorded. Toxicity was assessed using the CTCAE 5.0.

### Statistics

The primary outcome was overall survival after HSRS, defined as survival from the time of the completion of HSRS to death due to any cause. Other measures included progression-free survival after salvage treatment and treatment-related toxicities.

Survival curves were estimated using the Kaplan-Meier method and compared with the log-rank test.

Multivariate analysis of OS-HSRS in WHO grade 4 glioma patients was performed using a Cox proportional hazards regression model. Variables included in the multivariate analysis model were those with hypotheses of interest or determined to be clinically related to survival. Age, PTV, biologically effective dose (the median BED was 37.5 Gy, BED was calculated using the LQ model with an alpha/beta ratio of 10, and KPS and concurrent bevacizumab regimen (defined as bevacizumab administered during HSRS) were factors included in the multivariate analysis. The multivariable Cox regression model results are reported as hazard ratios with 95% CI and *p* values. All statistical analyses were performed in R version 3.6.1 using the survminer and survival packages.

## Results

### Patient characteristics

**Fig. 1:**
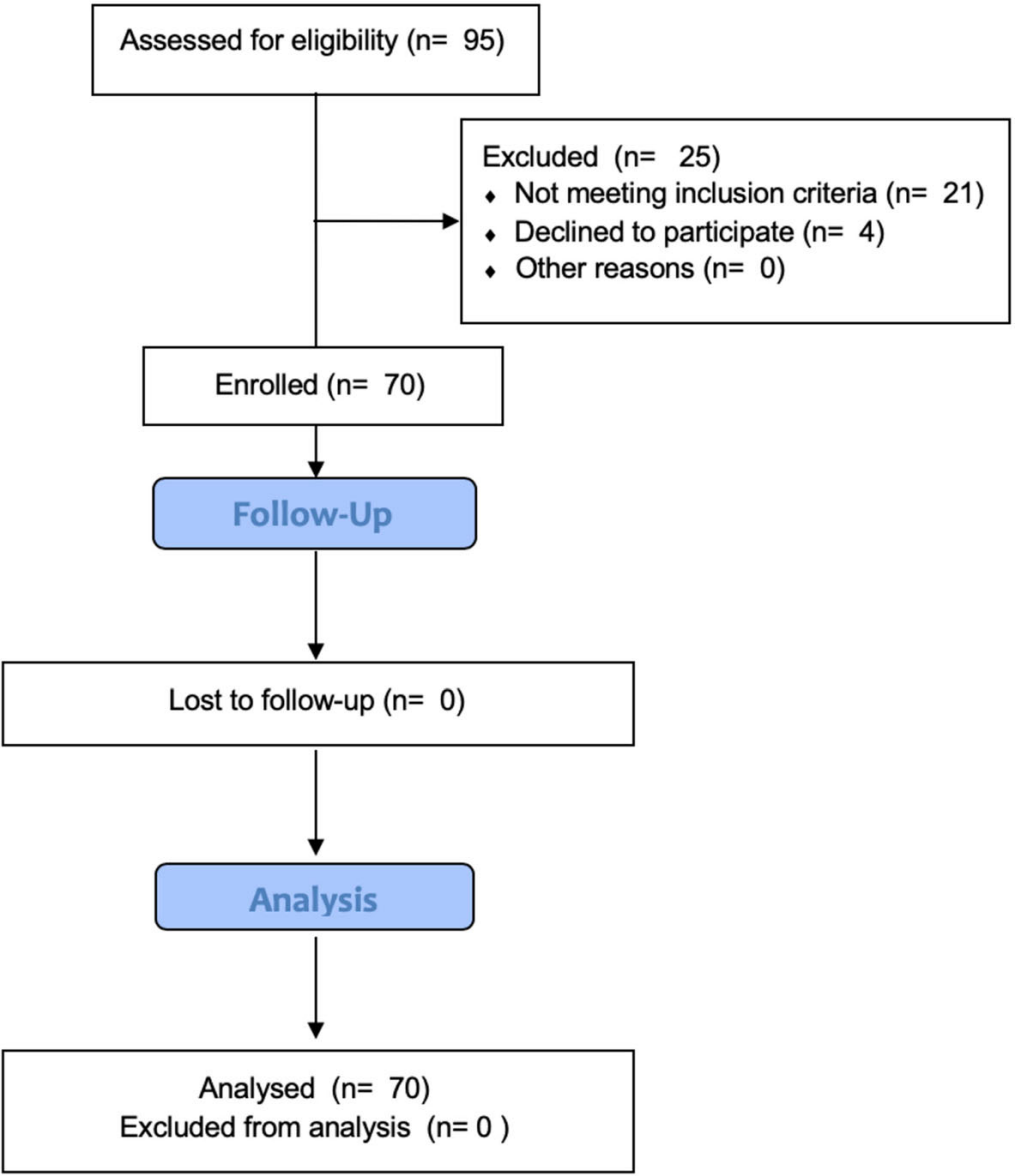
Flow diagram of this retrospective study

Between January 2016 and October 2019, 70 high-grade glioma patients who had clinical, radiographic and/or stereotactic brain biopsy evidence of recurrence were treated with CyberKinfe (Fig. 1). All patients were initially treated with maximum safe resection and adjuvant radiation treatment with a median dose of 60 Gy in 30 fractions with concurrent and maintenance temozolomide. Forty patients were male and 30 were female. The median age was 53 years (range 20–76 years). The majority of patients (49) had an initial diagnosis of glioblastoma, and the rest (21) had WHO grade 3 gliomas. The median time from the initial diagnosis to salvage HSRS was 13.7 months, with a range of 4.2 to 55.3 months.

The median volume of salvage HSRS PTV was 16.68 cm^3^ (0.81–121.96 cm^3^). The treatment was given daily, and the median dose was 24 Gy (12–30 Gy) in 4 fractions (2–6 fractions), with a median isodose line of 70% (63–75%). Patient characteristics are listed in Table 1.

**Table 1:** Baseline Characteristics of All Patients (*N* = 70)

Parameter	*N*	%
Gender		
Male	40	57
Female	30	43
Age, years		
Median	53	/
Range	20–76	/
Initial histological WHO grade		
3	21	30
4	49	70
Interval between HSRS and initial diagnosis (months)		
Median	13.7	/
Range	1.1–55.3	/
KPS before salvage HSRS		
Median	70	/
Range	40–90	
MGMT		
Unmethylated	25	36
Methylated	9	13
Unknown	36	51
IDH1 mutation		
Yes	5	7
No	45	64
Unknown	20	29
1p19q codeletion		
Yes	1	1
No	25	36
Unknown	44	63
Planning tumour volume (cm^3^)		
Median	16.68	/
Range	0.81–121.96	/
Concurrent systemic therapy		
TMZ	14	20
BVZ	28	40
TMZ + BVZ	7	10
BSC	21	30
Total	70	100

*Abbreviations*: *WHO* World Health Organization, *HSRS* hypofractionated stereotactic radiosurgery, *KPS* Karnofsky performance status, *MGMT* O6-methylguanine-DNA methyltransferase, *IDH1* Isocitrate dehydrogenase 1, *TMZ* Temozolomide, *BVZ* Bevacizumab, *BSC* best supportive care

### Compliance and toxicities

All patients completed planned HSRS without interruption. There was no significant treatment-related acute toxicity (grade ≥ 3). Common nonhaematologic grade 2 toxicities included fatigue (9 cases, 12.9%), nausea/vomiting (7 cases, 10.0%) and headache (4 cases, 5.7%). Toxicity details are shown in Table 2.

**Table 2:** Adverse Events Occurring in rHGG Patients

Adverse Events	Total No. of Patients	No. of Patients		
		Grade 1	Grade 2	Grade 3/4
Haematologic				
Anaemia	13	8	5	0
Neutropenia	11	5	6	0
Lymphocytopenia	7	2	5	0
Thrombocytopenia	7	3	4	0
Nonhaematologic				
Fatigue	32	23	9	0
Hypertension	21	18	3	0
Headache	21	17	4	0
Nausea and vomiting	13	6	7	0
Seizure	7	7	0	0
AST^*^ increased	5	2	3	0
ALT^†^ increased	4	1	3	0

*Abbreviations*: *AST* aspartate aminotransferase, *ALT* alanine aminotransferase

Other toxicities included hypertension, seizure and haematologic toxicities that were considered chemotherapy related. No complications were related to acute or late toxicity of HSRS.

### Treatment outcomes

**Fig. 2:**
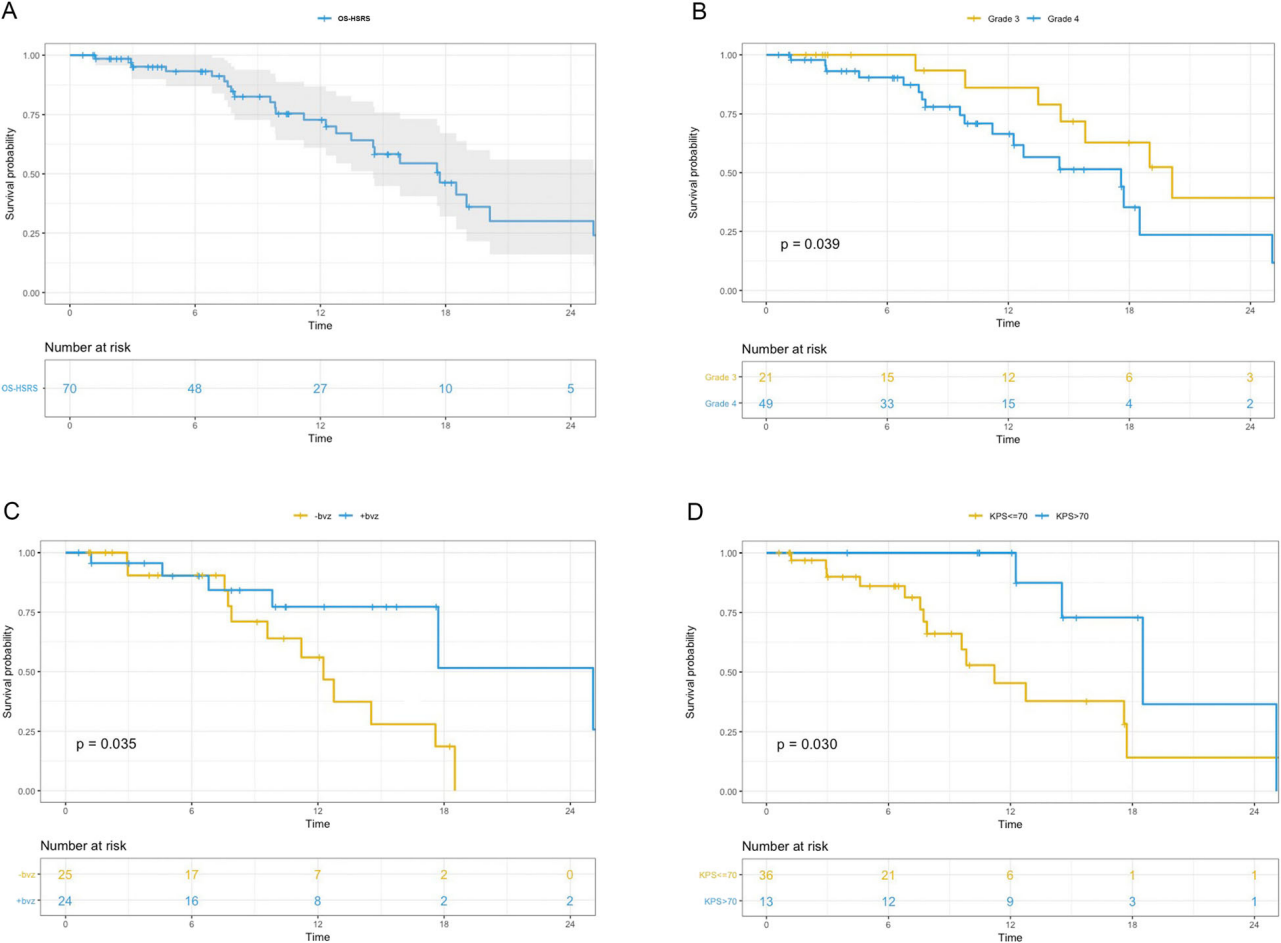
**a** Survival time from salvage HSRS (OS-HSRS) of all rHGG patients; **b** OS-HSRS for WHO Grade 3 and 4 patients; **c** OS-HSRS for WHO Grade 4 patients who underwent concurrent bevacizumab with HSRS; **d** OS-HSRS for WHO Grade 4 patients who had KPS ≤ 70 v > 70

By the end of the study, 26 patients died of tumour progression. The median follow-up from the time of HSRS was 12.1 months. The median overall survival after salvage treatment was 17.6 months for the whole cohort (Fig. 2a, 14.5 to 26.1 months, 95% CI) and 19.5 and 14.6 months for grade 3 and 4 gliomas, respectively (Fig. 2b, p = 0.039). The overall survival rates following HSRS were 72.8, 30.1, and 18.0% at 1, 2, and 3 years, respectively.

All patients were assessed by the RANO criteria, and 42 patients had progressive disease. Twelve of the patients (28.6%) had new lesions outside the radiation field, and 30 patients (71.4%) had local recurrence (LR). The 6-month PFS was 76.3 and 57.1% for grade 3 and 4 patients, respectively. The median PFS was 7.0 months (5.9 to 9.2 months, 95% CI) and 7.6 and 6.8 months for grades 3 and 4, respectively (*p* = 0.077). Twenty-four patients received a second-course salvage treatment because of radiographic progression, and two of them received surgery.

On multivariate analysis, KPS > 70 (HR = 0.13, *p* = 0.0060) and bevacizumab concurrently administered with HSRS (defined as bevacizumab administered during HSRS, HR = 0.15, *p* = 0.0040) were factors that positively affected OS after salvage HSRS for grade 4 patients (Table 3). The one-year OS rates after HSRS in grade 4 patients who had concurrent bevacizumab with HSRS and those who did not were 77.3 and 56.0%, respectively (Fig. 2c, p = 0.035). Grade 4 patients with a KPS > 70 before treatment also showed a longer OS than those with a KPS ≤ 70 (Fig. 2d, p = 0.041).

**Table 3:** Multivariate Analysis of Survival after Salvage Treatment in Grade 4 rHGG Patients

Factors	Comparison	HR	95%CI	*P*
Age, years	Continuous	1.03	0.98–1.08	0.250
KPS	> 70 v ≤ 70	0.13	0.03–0.56	0.006
PTV, cm^3^	> 10 v ≤ 10	0.44	0.13–1.50	0.187
BED, Gy	> 37.5 v ≤ 37.5	1.50	0.53–4.27	0.446
Concurrent^†^ bevacizumab	Yes v no	0.15	0.04–0.56	0.004

*Abbreviations*: *HR* hazard ratio, *CI* confidence interval, *KPS* Karnofsky performance status, *PTV* planning target volume, *BED* biologically effective dose

*BED was calculated using the LQ model with an alpha/beta ratio of 10

†Concurrent was defined as bevacizumab administered with HSRS

There was no significant difference in age, sex, KPS, mutation status, time from the initial diagnosis to HSRS or PTV between these two groups.

## Discussion

### Salvage treatment options for recurrent high-grade Glioma

For rHGG patients, salvage treatment included surgery, reirradiation, systemic therapy, and TTF. Although the results of prospective trials on these regimens have been published (Table 4), no standard treatment exists [9, 12, 16]. Thus, an individualized option that considers efficacy, quality of life and toxicity is crucial.

**Table 4:** Recent Prospective Trials of Recurrent High-grade Glioma

Treatment	Author, Year	Regimen	Median Dose(Gy)	Sample Size(N)	MST (Months)
Systemic Therapy	Reardon, 2017 [8]	Nivolumab vs BVZ	N/A	Nivo = 184 BVZ = 185	9.8 vs 10.0
	Wick, 2017 [9]	Lomustine +BVZ vs Lomustine	N/A	L + BVZ = 228 L = 149	9.1 vs 8.6
	Friedman, 2009 [10]	BVZ vs BVZ + CPT11	N/A	BVZ = 85 BVZ + CPT11 = 82	9.2 vs 8.7
TTF	Stupp, 2012 [12]	TTField vs Chemotherapy	N/A	TTF = 120 Chemotherapy = 117	6.6 vs 6
HSRT	Clarke, 2017 [13]	HSRT+BVZ	30	15	12.5
	Miwa, 2014 [14]	HSRT+TMZ	30	21	12.0
	Wuthrick, 2014 [15]	HSRT+Sunitinib	35	11	12.7(GBM)

*Abbreviations*: *TTF* Tumor Treatment Field, *HSRT* Hypofractionated stereotactic radiotherapy, *BVZ* Bevacizumab, *TMZ* Temozolomide, *GBM* Glioblastoma Multiforme

The efficacy of systemic therapy in improving OS for rHGG is unclear. Bevacizumab improved PFS with a median OS of 9 months in two randomized controlled trials (RCTs) [9, 10]. Programmed death-1 (PD-1) immune checkpoint inhibitor antibody showed a negative result compared with bevacizumab, with a median OS of 9.8 months and median PFS of 1.5 months in the phase III RCT Checkmate 143 trial [8].

HSRS and hypofractionated stereotactic radiotherapy (HSRT) using linear accelerators take advantage of the stereotactic precision as well as the properties of a standard fractionation schedule. Irradiated recurrent tumours allow a high treatment dose to cover the PTV and minimize normal brain toxicity. In addition, the condensed treatment schedule could be an important option for rHGG patients with short expected survival and poor KPS. HSRT has been reported to have a median overall survival for rHGG patients ranging from 12 to 12.7 months. Conti et al. reported a median survival of 12 months for recurrent glioblastoma patients who underwent HSRS reirradiation with temozolomide 75 mg/m2/day for 21 days every 28 days [17]. The prospective trial by Wuthrick reported a median survival of 12.7 months in grade 4 glioma patients using HSRT of 30 to 42 Gy in 2.5 to 3.75 Gy fractions with 37.5 daily sunitinib [15]. Fogh et al. reported the largest series retrospective study of 147 patients using X-knife with a median dose of 35 Gy in 10 fractions. The median survival achieved 11 months [18]. Additionally, Shi et al. reported a cohort of 36 grade 2 to 4 glioma patients using 30–35 Gy/10 fx HSRT with alisertib who achieved a median overall survival of 11.1 months [19].

There are limited data addressing the combination of systematic therapy and reirradiation for rHGG [20]. Several prospective trials examined the safety and efficacy of HSRT with systematic therapy for rHGG exhibiting OS ranging from 12 months to 12.7 months (Table 4). Minniti et al. examined HSRT with TMZ in 54 rHGG patients. With 30 Gy in 5 fractions plus concomitant TMZ up to 12 cycles, the median survival after salvage treatment was 12.4 months. KPS > 70 and grade 3 glioma were considered prognostic factors for survival [16].

### Effectiveness of CyberKnife re-irradiation for rHGG

CyberKnife is an image-guided stereotactic radiosurgery system that can deliver accurate treatment doses to brain lesions. In this study, we showed a median OS after HSRS of 17.6 months (19.5 and 14.6 months for grade 3 and 4 gliomas, respectively; *p* = 0.039). In the literature, the survival of grade 4 patients after CyberKnife was reported to range from 10.6 to 13.7 months [21, 22]. The promising survival in this study is due to several reasons. First, radiation was delivered with a relatively low isodose line of 63 to 75% and resulted in a higher dose delivered to the tumour. It increased the tumour centre dose and enhanced the tumour cell killing activity as a direct result as Romanelli et al. reported that the normalized total dose can achieve 57 Gy (24 Gy in 3 fractions 80% isodose) for the tumour using an α/β = 10 linear quadratic model [23]. Second, all the enrolled patients received HSRS as first-line salvage treatment. For these patients, survival was expected to be longer. Third, 24 (34.2%) patients underwent a second-course salvage treatment after HSRS, including surgery, HSRS, systemic therapy, and TTF.

Minimizing radiation injury to the normal brain was considered when increasing the treatment dose. Bevacizumab is an anti-vascular endothelial growth factor (VEGF) monoclonal antibody that is used in recurrent glioblastoma [9, 10]. Bevacizumab has been hypothesized to protect the normal brain from radiation by reducing brain oedema and radiation necrosis. The advantage of adding bevacizumab to HSRS has not been fully illustrated. Philip el al theorized that the additional bevacizumab sensitized the tumour endothelia to radiotherapy and induced apoptosis [24]. Additionally, Kyle et al. concluded that the perivascular niche and antitumour effects could be the reason.

Our data suggest that HSRS with concomitant bevacizumab and good performance status result in improved survival in grade 4 patients. These results are similar to Sharma’s research that reported 53 GBM patients who achieved a median survival of 11 months after gamma knife radiosurgery, and radiosurgery was associated with longer survival in good performance patients [25]. Additionally, Cuneo reported an OS of 11.2 months in patients receiving bevacizumab and stereotactic radiosurgery compared with 3.9 months for patients treated with stereotactic radiosurgery alone [26]. However, the preliminary results of RTOG1205 showed a negative result of HSRT in improving OS [27]. A variety of radiotherapy techniques used in the trial and a relatively larger median PTV may contaminate the result. However, an improved 6-month PFS was achieved in the HSRT+BVZ group compared with the BVZ only group, and a longer PFS can increase the quality of life in brain tumour patients. For rHGG patients who have few or other therapeutic options, HSRS or HSRT combined with bevacizumab may represent a reasonable consideration.

### Strengths and limitations

To date, this is the largest cohort of CyberKnife as a first-line salvage treatment for recurrent high-grade glioma within radiation field patients. Our study demonstrates promising survival and mild toxicity using CyberKnife radiosurgery for rHGG patients.

However, the retrospective nature limited this study. Selection bias was created when deciding the eligible patients for salvage treatment, which increased the number of potential good prognosis patients. Additionally, additional systemic therapy, second-course salvage treatment after HSRS and lack of imaging follow-up for palliative care patients may influence the result. Moreover, both previous studies and clinical experience at our centre [see Additional file 2] encountered the same dilemma as irradiated brain tumours, and the diagnosis of LR and RN was difficult [28, 29].

Despite the limitations, this study presented a promising outcome of salvage HSRS. A prospective phase II study HSCK-002 ClinicalTrials.gov identifier, NCT04197492, is ongoing at our centre to further investigate the value of HSRS and anlotinib (an oral novel multitarget tyrosine kinase inhibitor targeting VEGF receptor, fibroblast growth factor receptor and platelet-derived growth factor receptor).

## Conclusions

HSRS using the CyberKnife radiosurgery system showed a favourable outcome and acceptable toxicity as a salvage treatment for HGG at first recurrence. A prospective phase II trial, HSCK-002 (ClinicalTrials.gov identifier: NCT04197492), is ongoing to further evaluate the efficacy of CyberKnife radiosurgery for rHGG.

## Supplementary Information

**Additional file 1.** Treatment of progression after CyberKnife radiosurgery. This file gives the details of therapy to all the participants.

**Additional file 2.** The dilemma of the diagnosis of LR and RN. This pdf shows two cases in our center indicating the difficulty in diagnosing the local recurrence of HGG and radiation neurosis.

## Data Availability

The datasets used and/or analyzed in this study are available from the corresponding author on request.

## Abbreviations

rHGG: Recurrent high-grade gliomas
PFS: Progression-free survival
GBM: Glioblastoma
PTV: Planning target volume
KPS: Karnofsky Performance Status
OS: Overall survival
WHO: World Health Organization
GTV: Gross tumor volume
CTV: Clinical tumor volume
BED: Biologically effective dose
LR: Local recurrence
HSRS: Hypofractionated stereotactic radiosurgery
RCT: Randomized control trials
PD-1: Programmed death-1
HSRT: Hypofractionated stereotactic radiotherapy

## References

[1] Jemal A, Siegel R, Ward E (2006). Cancer statistics, 2006. CA Cancer J Clin.

[2] Stupp R, Mason WP, van den Bent MJ (2005). Radiotherapy plus concomitant and adjuvant temozolomide for glioblastoma. N Engl J Med.

[3] Kirkpatrick JP, Sampson JH (2014). Recurrent malignant gliomas. Semin Radiat Oncol.

[4] Martínez-Carrillo M, Tovar-Martín I, Zurita-Herrera M, et al. Salvage radiosurgery for selected patients with recurrent malignant gliomas. Biomed Res Int. 2014:657953. 10.1155/2014/657953 PMC4033521 24895599

[5] Montemurro N, Perrini P, Blanco MO (2016). Second surgery for recurrent glioblastoma: a concise overview of the current literature. Clin Neurol Neurosurg.

[6] Barbagallo GMV, Jenkinson MD, Brodbelt AR (2008). Recurrent glioblastoma multiforme, when should we reoperate?. Br J Neurosurg.

[7] Park JK, Hodges T, Arko L (2010). Scale to predict survival after surgery for recurrent glioblastoma multiforme. J Clin Oncol.

[8] Reardon DA, Omuro A, Brandes AA (2017). OS10. 3 randomized phase 3 study evaluating the efficacy and safety of nivolumab vs bevacizumab in patients with recurrent glioblastoma: CheckMate 143. Neuro-oncology.

[9] Wick W, Gorlia T, Bendszus M (2017). Lomustine and bevacizumab in progressive glioblastoma. N Engl J Med.

[10] Friedman HS, Prados MD, Wen PY (2009). Bevacizumab alone and in combination with irinotecan in recurrent glioblastoma. J Clin Oncol.

[11] Wallner KE, Galicich JH, Krol G (1989). Patterns of failure following treatment for glioblastoma multiforme and anaplastic astrocytoma. Int J Radiat Oncol Biol Phys.

[12] Stupp R, Wong ET, Kanner AA (2012). NovoTTF-100A versus physician’s choice chemotherapy in recurrent glioblastoma: a randomised phase III trial of a novel treatment modality. Eur J Cancer.

[13] Clarke J, Neil E, Terziev R (2017). Multicenter, phase 1, dose escalation study of hypofractionated stereotactic radiation therapy with bevacizumab for recurrent glioblastoma and anaplastic astrocytoma. Int J Radiat Oncol Biol Phys.

[14] Miwa K, Matsuo M, Ogawa S (2014). Re-irradiation of recurrent glioblastoma multiforme using 11 C-methionine PET/CT/MRI image fusion for hypofractionated stereotactic radiotherapy by intensity modulated radiation therapy. Radiat Oncol.

[15] Wuthrick EJ, Curran WJ, Camphausen K (2014). A pilot study of hypofractionated stereotactic radiation therapy and sunitinib in previously irradiated patients with recurrent high-grade glioma. Int J Radiat Oncol Biol Phys.

[16] Minniti G, Scaringi C, De Sanctis V (2013). Hypofractionated stereotactic radiotherapy and continuous low-dose temozolomide in patients with recurrent or progressive malignant gliomas. J Neuro-Oncol.

[17] Conti A, Pontoriero A, Arpa D (2012). Efficacy and toxicity of CyberKnife re-irradiation and “dose dense” temozolomide for recurrent gliomas. Acta Neurochir.

[18] Fogh SE, Andrews DW, Glass J (2010). Hypofractionated stereotactic radiation therapy: an effective therapy for recurrent high-grade gliomas. J Clin Oncol.

[19] Wenyin S, Erik S Blomain, Joshua Siglin, et al. (2018). Salvage fractionated stereotactic re-irradiation (FSRT) for patients with recurrent high-grade gliomas progressed after bevacizumab treatment. J Neuro-Oncol.

[20] Yazici G, Cengiz M, Ozyigit G (2014). Hypofractionated stereotactic reirradiation for recurrent glioblastoma. J Neuro-Oncol.

[21] Pinzi V, Orsi C, Marchetti M (2015). Radiosurgery reirradiation for high-grade glioma recurrence: a retrospective analysis. Neurol Sci.

[22] Yersal Ö (2017). Clinical outcome of patients with glioblastoma multiforme: single center experience. J Oncol Sci.

[23] Romanelli P, Conti A, Pontoriero A (2009). Role of stereotactic radiosurgery and fractionated stereotactic radiotherapy for the treatment of recurrent glioblastoma multiforme. Neurosurg Focus.

[24] Gutin PH, Iwamoto FM, Beal K (2009). Safety and efficacy of bevacizumab with hypofractionated stereotactic irradiation for recurrent malignant gliomas. Int J Radiat Oncol Biol Phys.

[25] Sharma M, Schroeder JL, Elson P (2018). Outcomes and prognostic stratification of patients with recurrent glioblastoma treated with salvage stereotactic radiosurgery. J Neurosurg.

[26] Cuneo KC, Vredenburgh JJ, Sampson JH (2012). Safety and efficacy of stereotactic radiosurgery and adjuvant bevacizumab in patients with recurrent malignant gliomas. Int J Radiat Oncol Biol Phys.

[27] Tsien C, Pugh S, Dicker AP (2019). Randomized phase II trial of re-irradiation and concurrent Bevacizumab versus Bevacizumab alone as treatment for recurrent Glioblastoma (NRG oncology/RTOG 1205): initial outcomes and RT plan quality report. Int J Radiat Oncol Biol Phys.

[28] Yomo S, Hayashi M (2016). Salvage stereotactic radiosurgery with adjuvant use of bevacizumab for heavily treated recurrent brain metastases: a preliminary report. J Neuro-Oncol.

[29] Guan Y, Wang C, Zhu H (2020). Hypofractionated radiosurgery plus Bevacizumab for locally recurrent brain metastasis with previously high-dose irradiation. World Neurosurg.

