# Supplementary material for: Safety and efficacy of Hypofractionated stereotactic radiosurgery for high-grade Gliomas at first recurrence: a single-center experience
Source: BMC Cancer. 2021 Feb 5;21:123. doi: 10.1186/s12885-021-07856-y (PMC7863415; doi:10.1186/s12885-021-07856-y)
Supplement: Supplementary file 1 — Additional file 1. Treatment of progression after CyberKnife radiosurgery. This file gives the details of therapy to all the participants. [file 12885_2021_7856_MOESM1_ESM.pdf]

Title Page

Title: Safety and Efficacy of Hypofractionated Stereotactic Radiosurgery for High-grade Gliomas at First Recurrence: A Single-Center Experience

Running Title: CyberKnife for Recurrent High-grade Gliomas

Authors: Yun Guan[1234*] MD, Ji Xiong[5] MD, Mingyuan Pan[1234*] M.S., Wenyin Shi[6] PhD, Jing Li[1234] MD, Huaguang Zhu[1234] MD, Xiu Gong[1234] MD, Chao Li[1234] MD, Guanghai Mei[1234] MD, Xiaoxia Liu[1234] MD, Li Pan[1234] MD, Jiazhong Dai[1234] MD, Yang Wang[1234] MD, Enmin Wang[1234&] MD, Xin Wang[1234&] MD

1 CyberKnife Center, Department of Neurosurgery, Huashan Hospital, Fudan University, 12 Wulumuqi Road (M), Shanghai, 200040, China.

2 Neurosurgical Institute of Fudan University, 12 Wulumuqi Road (M), Shanghai, 200040, China.

3 Shanghai Clinical Medical Center of Neurosurgery, 12 Wulumuqi Road (M), Shanghai, 200040, China.

4 Shanghai Key Laboratory of Brain Function and Restoration and Neural Regeneration, 12 Wulumuqi Road (M), Shanghai, 200040, China.

5 Department of pathology, Huashan Hospital, Fudan University, 12 Wulumuqi Road (M), Shanghai, 200040, China.

6 Department of Radiation Oncology, Thomas Jefferson University, Philadelphia, PA, USA.

&Corresponding author:

Xin Wang, MD

E-mail: wangxinck@fudan.edu.cn

Tel: +86-13120557273

Address: CyberKnife Center, Department of Neurosurgery, Huashan Hospital, Fudan University, 12 Wulumuqi Road (M), Shanghai, 200040, China.

Enmin Wang, MD

E-mail: emwang@fudan.edu.cn

Tel: +86-13671727320

Address: CyberKnife Center, Department of Neurosurgery, Huashan Hospital, Fudan University, 12 Wulumuqi Road (M), Shanghai, 200040, China.

Additional file 1. Treatment of progression after CyberKnife radiosurgery

Treatment		No. of Patients	Percentage %
Surgery		2	3
Radiotherapy			
	IMRT*	1	1
	CyberKnife	13	19
TTF†		2	3
Systemic therapy			
	Anti-PD-1	3*	4
	Anlotinib	2	3
	Bevacizumab	1	1
	Temozolomide	1	1
BSC‡		46	65

Abbreviations: IMRT = Intensity-modulated radiation therapy ; TTF = Tumor Treatment Field; BSC = Best Supportive Care.

* One patient had CyberKnife with anti-PD-1 treatment.

Additional file 2. The dilemma of diagnosis of the LR and RN

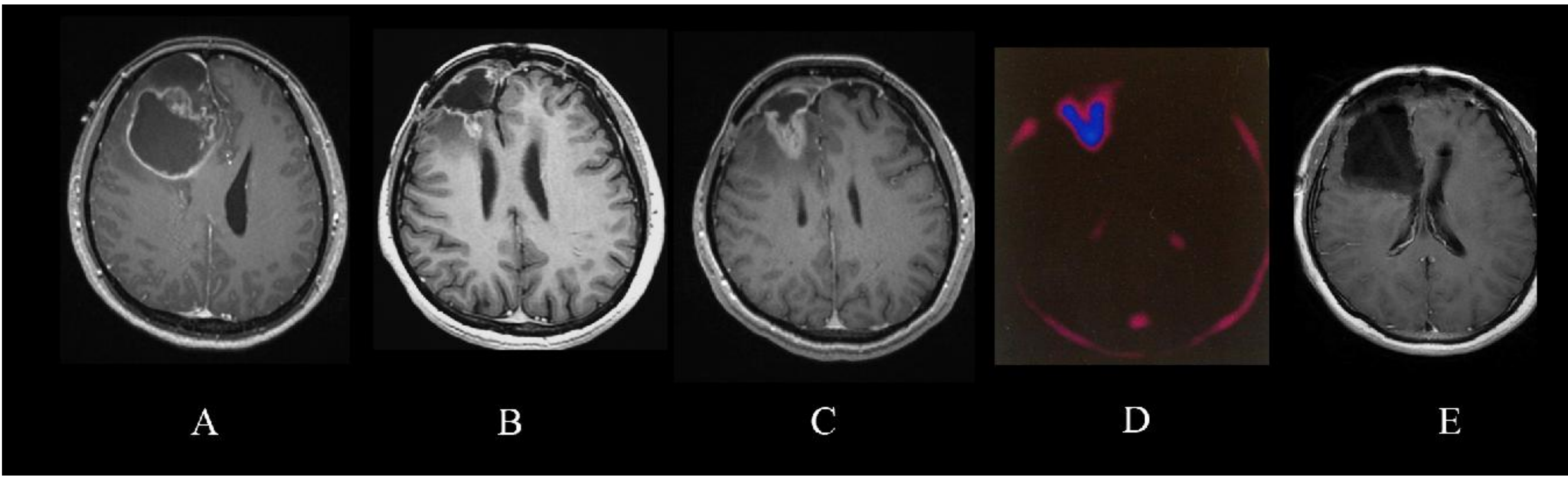

Case 1.

A. Forty-year-old female, presented with headache and dizziness. Histological-proven glioblastoma, MGMT (-), IDH1(+), 1p19q (-).

B. RANO criteria diagnosed recurrence. Seventeen months after surgery and adjuvant chemoradiation IMRT 56Gy/28fx and temozolomide.

C, D. Recurrence diagnosed by RANO criteria and 11C-methionine PET. Six months after CyberKnife 20Gy/5fx with concurrent bevacizumab.

E. Histological-proven radiation necrosis after gross total resection.

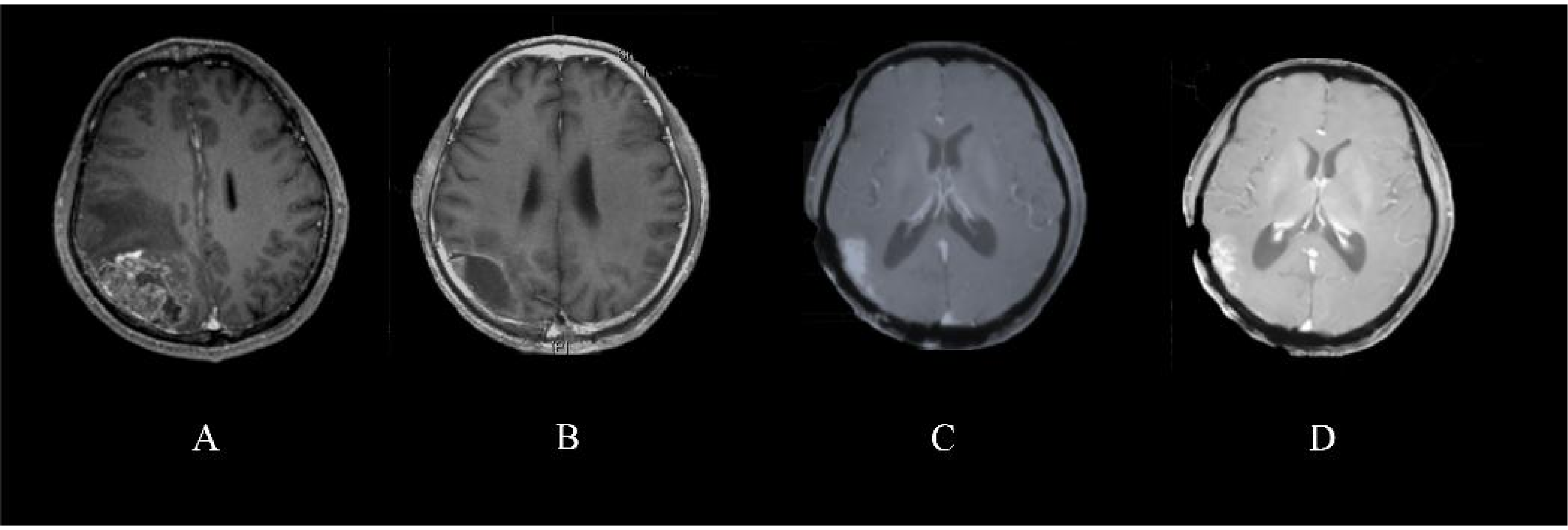

Case 2.

A. Fifty-eight years old male. Histological-proven glioblastoma, MGMT (-), IDH1(-), 1p19q (-), TERT (+).

B. Treated with surgery and adjuvant chemoradiation IMRT 60Gy/30fx plus temozolomide and TTF. Four months after initial diagnosis.

C. Twelve months after surgery, RANO criteria diagnosed recurrence.

D. Three months follow-up after CyberKnife 25Gy/5fx with concurrent bevacizumab.
