# Supplementary material for: Safety and efficacy of Hypofractionated stereotactic radiosurgery for high-grade Gliomas at first recurrence: a single-center experience
Source: BMC Cancer. 2021 Feb 5;21:123. doi: 10.1186/s12885-021-07856-y (PMC7863415; doi:10.1186/s12885-021-07856-y)
Supplement: Supplementary file 2 — Additional file 2. The dilemma of the diagnosis of LR and RN. This pdf shows two cases in our center indicating the difficulty in diagnosing the local recurrence of HGG and radiation neurosis. [file 12885_2021_7856_MOESM2_ESM.docx]

Additional file 2. The dilemma of diagnosis of the LR and RN


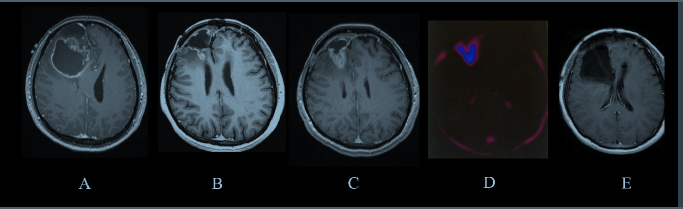


Case 1.

A. Forty-year-old female, presented with headache and dizziness. Histological-proven

glioblastoma, MGMT (-), IDH1(+), 1p19q (-).

B. RANO criteria diagnosed recurrence. Seventeen months after surgery and adjuvant

chemoradiation IMRT 56Gy/28fx and temozolomide.

C, D. Recurrence diagnosed by RANO criteria and 11C-methionine PET. Six months after

CyberKnife 20Gy/5fx with concurrent bevacizumab.

E. Histological-proven radiation necrosis after gross total resection


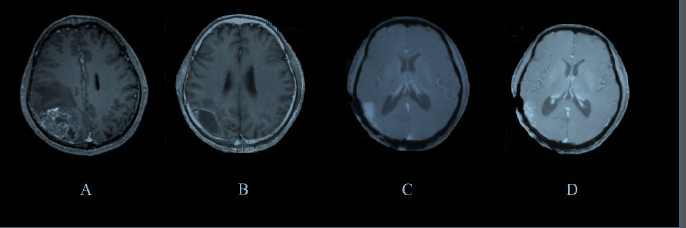


Case 2.

A. Fifty-eight years old male. Histological-proven glioblastoma, MGMT (-), IDH1(-), 1p19q (-),

TERT (+).

B. Treated with surgery and adjuvant chemoradiation IMRT 60Gy/30fx plus temozolomide and

TTF. Four months after initial diagnosis.

C. Twelve months after surgery, RANO criteria diagnosed recurrence.

D. Three months follow-up after CyberKnife 25Gy/5fx with concurrent bevacizumab.
